# Antitumor activity of a newly developed monoclonal antibody against ROR1 in ovarian cancer cells

**DOI:** 10.18632/oncotarget.21618

**Published:** 2017-10-07

**Authors:** Zhengna Yin, Mengyun Gao, Sasa Chu, Yiping Su, Chunping Ye, Yiquan Wang, Zhuanqin Pan, Zhuming Wang, Huilin Zhang, Hua Tong, Jin Zhu

**Affiliations:** ^1^ Department of Obstetrics and Gynecology, Nanjing Maternity and Child Health Care Hospital, Obstetrics and Gynecology Hospital Affiliated to Nanjing Medical University, Nanjing 210004, China; ^2^ Department of Infectious Disease, Institute of Liver Disease, Nanjing Jingdu Hospital, Nanjing 210002, China; ^3^ Department of Traditional Chinese Internal Medicine, Longhua Hospital Affiliated to Shanghai University of Traditional Chinese Medicine, Shanghai 200071, China; ^4^ Department of Nursing, Gaoyou People’s Hospital, Yangzhou 225600, China; ^5^ Department of Pathology, Chinese Ministry of Health-designated Key Laboratory of Antibody Technology, Nanjing Medical University, Nanjing 210029, China; ^6^ Huadong Medical Institute of Biotechniques, Nanjing 210002, China

**Keywords:** ROR1, monoclonal antibody production, chimeric antibody Fab, binding affinity, antitumor activity

## Abstract

Receptor-tyrosine-kinase-like Orphan Receptor 1 (ROR1) is a tyrosine-protein kinase transmembrane receptor and ROR1 overexpression is associated with a poor prognosis in various cancers, including ovarian cancer. Targeting of ROR1 has been evaluated as a novel cancer therapy strategy. This study developed a novel chimeric anti-ROR1 Fab antibody (named ROR1-cFab) and then assessed the antitumor activity of this antibody in ovarian cancer cells, an *in vitro* model of preclinical cancer therapy. A ROR1-cFab prokaryotic expression vector was constructed from positive fusion cells (splenocytes from mice with high ROR1 immune titers were fused with myeloma cells) after three rounds of sub-clone affinity screening. Then, a variety of assays were employed to assess the binding selectivity and specificity of ROR1-cFab to ROR1 protein. Furthermore, CCK8, flow cytometric apoptosis, wound healing, and Transwell migration assays were used to assess antitumor activity of this newly developed anti-ROR1 antibody in ovarian cancer cells. We demonstrated that ROR1-cFab could specifically bind to ROR1 protein and ROR1-positive ovarian cancer A2780 cells. Functional assays revealed that ROR1-cFab inhibited tumor cell proliferation and migration, as well as inducing apoptosis of ROR1-positive A2780 cells in a dose dependent manner. These effects were not observed in ROR1-negative lose386 cells. In conclusion, ROR1-cFab is a novel anti-ROR1 antibody with a high affinity to ROR1 protein and inhibitory effects on ROR1-positive cells. Future studies will determine whether the ROR1-cFab might be a promising candidate for treatment of ROR1-positive ovarian cancer.

## INTRODUCTION

Receptor-tyrosine-kinase-like Orphan Receptor 1 (ROR1) is a tyrosine-protein kinase transmembrane receptor, which consists of FZ (frizzled), Ig-like C2-type (immunoglobulin-like), kringle, and protein kinase domains [1], and functions to regulate cell growth in the central nervous system during development [2, 3]. ROR1 has recently been shown to play an important role in metastasis [4]. Indeed, ROR1 expression was expressed in various human cancers [5–12] and considered as a biomarker for the prediction of the prognosis of various malignancies [7–9]. However, a recent study demonstrated that ROR1 protein is also expressed in certain healthy tissues, such as the parathyroid glands, pancreatic islets, esophagus, stomach, and duodenum [13]. Despite these findings, targeting of ROR1 has been suggested as a novel strategy for cancer therapy [14–17]. Indeed, as a family of proteins, receptor-tyrosine-kinases (RTKs) are known to participate in various cell functions, both physiologically and developmentally, such as cell proliferation, migration, survival, and differentiation [18–20]. Dysregulation of RTKs, including ROR1, has been demonstrated in various cancers and targeting of RTKs has served as a strategy for cancer treatment [21]. To target ROR1, a previous study reported that treatment of chronic lymphocytic leukemia (CLL) cells with monoclonal antibodies against ROR1 were able to induce apoptosis in tumor cells [22], while knockdown of ROR1 expression suppressed proliferation of lung adenocarcinoma cells [23]. In other malignancies, such as ovarian cancer, ROR1 has been shown to be highly expressed and may serve as a useful therapeutic target [14]. Indeed, targeting ROR1 suppressed both migration and invasion of epithelial ovarian cancer cells [24]. Inhibition of ROR1 expression using miR382 suppressed ovarian cancer cell migration and invasion by downregulating epithelial-mesenchymal transition [25]. Our previous study demonstrated that expression of ROR1 protein in ovarian cancer tissues was significantly higher than in normal ovary tissues and that ROR1 overexpression was associated with poor disease-free survival and overall survival [9].

To date, ovarian cancer remains the leading gynecological malignancy [26] with an increasing incidence and an overall poor survival rate. Despite noticeable improvement in early detection and treatment options, the majority of ovarian cancer patients are diagnosed at advanced stages of disease and often have a very poor prognosis [26]. Surgical approaches have been shown to cure early stage disease, however, platinum- and taxane-based drugs are most frequently used as first-line chemotherapy for advanced and unresectable ovarian cancers [27]. However, although ovarian cancer patients may initially well respond to these drugs or combinations, approximately 85% of these patients eventually relapse within a few years, including those who previously were deemed to have a complete response to treatment [28–30]. Thus, the search for and identification of biomarkers for the early detection of ovarian cancer, as well as the development of novel strategies to effectively treat ovarian cancer.

In our study, we first generated a novel monoclonal anti-ROR1 antibody by isolating chimeric anti-ROR1 Fab fragments (ROR1-cFab) from positive fusion cells after three rounds of sub-clone affinity screening and evaluation of affinity and binding to ROR1 protein and to ROR1-positive ovarian cancer cells. We then utilized ovarian cancer cells as an *in vitro* model to assess antitumor activity of our generated antibody in human cancer cells.

## RESULTS

### Generation of chimeric monoclonal antibody ROR1-cFab

**Figure 1:**
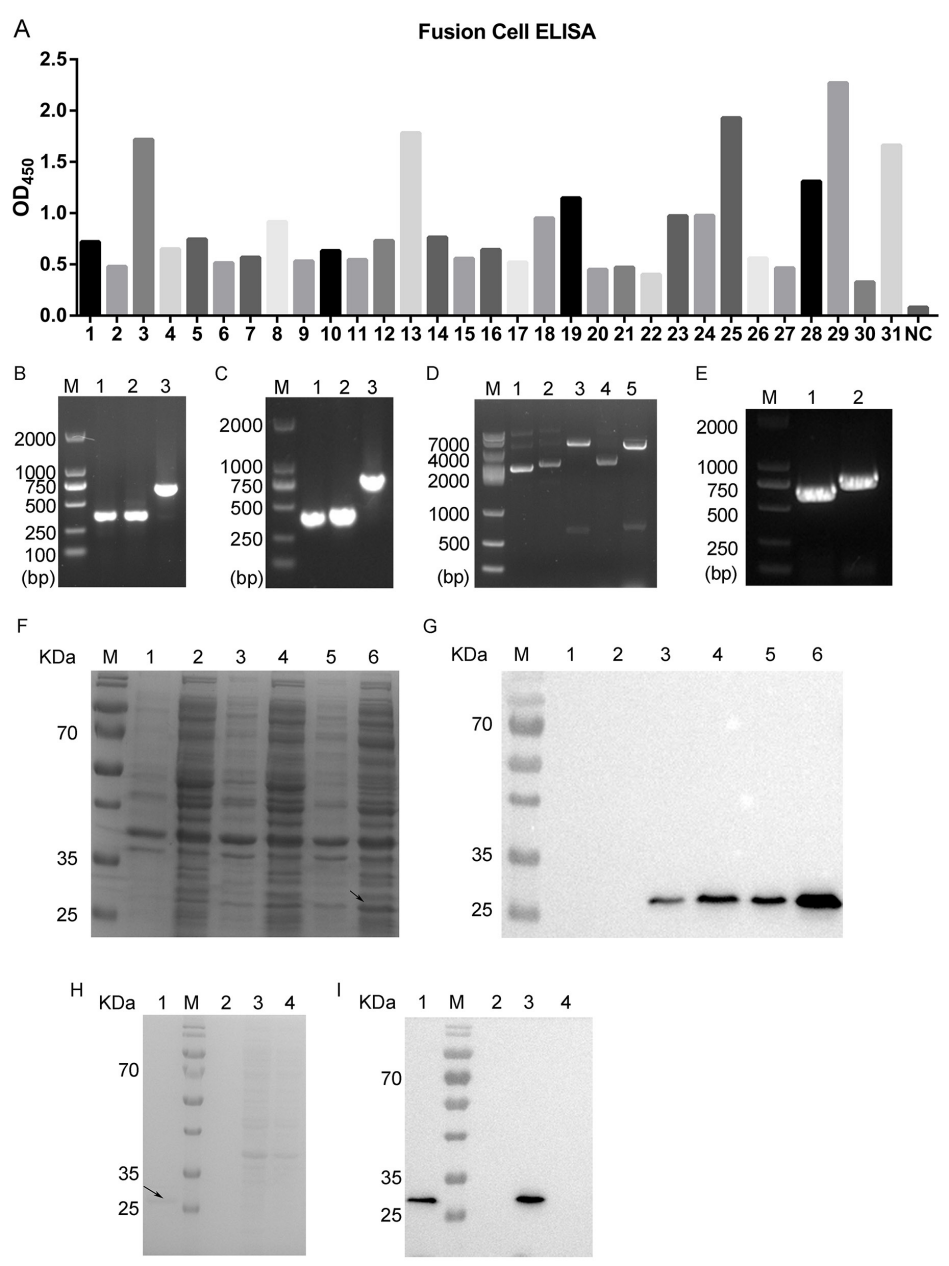
Screening and identification of positive fusion cell clones and generation of chimeric monoclonal antibody ROR1-cFab **(A)** ELISA. ELISA was performed to screen 31 candidate fusion cell clones after three rounds of sub-clone screening. NC, a negative control. **(B)** Agarose gels of PCR amplification of the light chain. Lane 1, V_L_; Lane 2, C_L_; Lane 3, V_L_ combined with C_L_; M, a DNA maker. **(C)** Agarose gels of PCR amplification of the heavy chain. Lane 1, V_H_; Lane 2, C_H_1; Lane 3, V_H_ combined with C_H_1; M, a DNA maker. **(D)** Agarose gels of PCR amplification of pETDuet-ROR1-cFab. Lane 1, the plasmid of pETDuet without restriction endonuclease digestion; Lane 2, the plasmid of pETDuet-L; Lane 3, NcoI/HindIII were used for double digesting the pETDuet-L plasmid; Lane 4, the plasmid of pETDuet-L-H (pETDuet-ROR1-cFab); Lane 5, NdeI/kpnI were used for double digestion of the pETDuet-ROR1-cFab plasmid; M, a DNA marker. **(E)** Agarose gels of PCR amplification of the light and heavy chain of pETDuet-ROR1-cFab. Lane 1, L; Lane 2, Fd; M, a DNA maker. **(F)** Coomassie blue staining of a SDS-PAGE gel and **(G)** Western blot. Detection of expression of the Fab fragment in E. coli. Lane 1, supernatant of sonicated lysate of untransfected E. coli BL21, a negative control; Lane 2, sediment of sonicated lysate of untransfected E. coli BL21, a negative control; Lane 3, supernatant of sonicated lysate of pETDuet-ROR1-cFab-transfected E. coli; Lane 4, sediment of sonicated lysate of pETDuet-ROR1-cFab-transfected E. coli; Lane 5, supernatant of sonicated lysate of pETDuet-ROR1-cFab-transfected E. coli induced by IPTG overnight; Lane 6, sediment of sonicated lysate of pETDuet-ROR1-cFab-transfected E. coli induced by IPTG overnight; M, a protein marker. **(H)** Coomassie blue staining and **(I)** Western blot. Detection of the purification efficiency of the Fab fragments. Lane 1, the Fab fragments was purified by the Protein L affinity column; lane 2, sediment of sonicated lysate of pETDuet-ROR1-cFab-transfected E. coli induced by IPTG overnight; lane 3, supernatant of sonicated lysate of pETDuet-ROR1-cFab-transfected E. coli induced by IPTG overnight; lane 4, the flow through the Protein L affinity column; M, a protein marker.

In this study, we first immunized mice with recombinant human ROR1 protein (Sino Biological Inc., Beijing, China) to isolate splenocytes with the highest immune ROR1 titers. These splenocytes were then fused with myeloma cells in order to screen for the positive fusion cell clones. Specifically, after three rounds of sub-clone affinity screening of myeloma hybrids, we obtained 40 positive fusion cell clones and then further analyzed using ELISA. Using the cut-off point of the ratio of sample versus the blank of more than four-fold we considered only these fused cell clones as candidates. Based on this, we determined 31 positive fusion cell clones and number 29 fusion cell clone was shown to possess the strongest binding ability to ROR1 protein (Figure 1A). We fully amplified five positive fused cell clones (#3, 13, 25, 29, and 31 fusion cells), analyzed and confirmed using DNA sequencing and then examined these against the VBASE2 database. We found that the light chain of the antibody was confirmed to be the kappa chain. Next, the V_L_ and V_H_ of positive clones were amplified and the length of V_L_, V_H_ and C_H_1 were approximately 400 bp of each. Similarly, the length of C_L_ obtained was approximately 350 bp (Figure 1B, 1C). After, we amplified the heavy chain Fd (800 bp) and the light chain L (750 bp) by using the overlap extension PCR according to a previous study [33]. DNA sequencing analysis verified that the Fd and L fragments were successfully inserted into the prokaryotic expression plasmid pETDuet-1 without any mutations (Figure 1D, 1E). Thus, we successfully constructed a prokaryotic expression vector carrying chimeric anti-ROR1 monoclonal Fab fragment (pETDuet-ROR1-cFab).

After *in vitro* analysis and Coomassie blue staining of the SDS-PAGE gel, the positive protein band was approximately 27 kDa, but due to the similar size, it was difficult for us to distinguish the Fd from the L band on the SDS-PAGE gel (Figure 1F). We thus performed a Western blot analysis using an antibody against the Fab fragments and found that the chimeric monoclonal antibody ROR1-cFab was mostly secreted in the ultrasonic supernatant but not in the ultrasonic sediment (Figure 1G).

Furthermore, considering the light chain of the Fab fragments was a kappa chain, we employed a protein L affinity column to purify the chimeric anti-ROR1 Fab fragments (ROR1-cFab) from the ultrasonic supernatant of the *E. coli* BL21 that was transformed by pETDuet-ROR1-cFab. We achieved approximately 95% purification efficiency for the Fab fragments, resulting in 900μg/mL of ROR1-cFab (Figure 1H, 1I).

### Verification of chimeric monoclonal antibody ROR1-cFab specifically binding to ROR1

**Figure 2:**
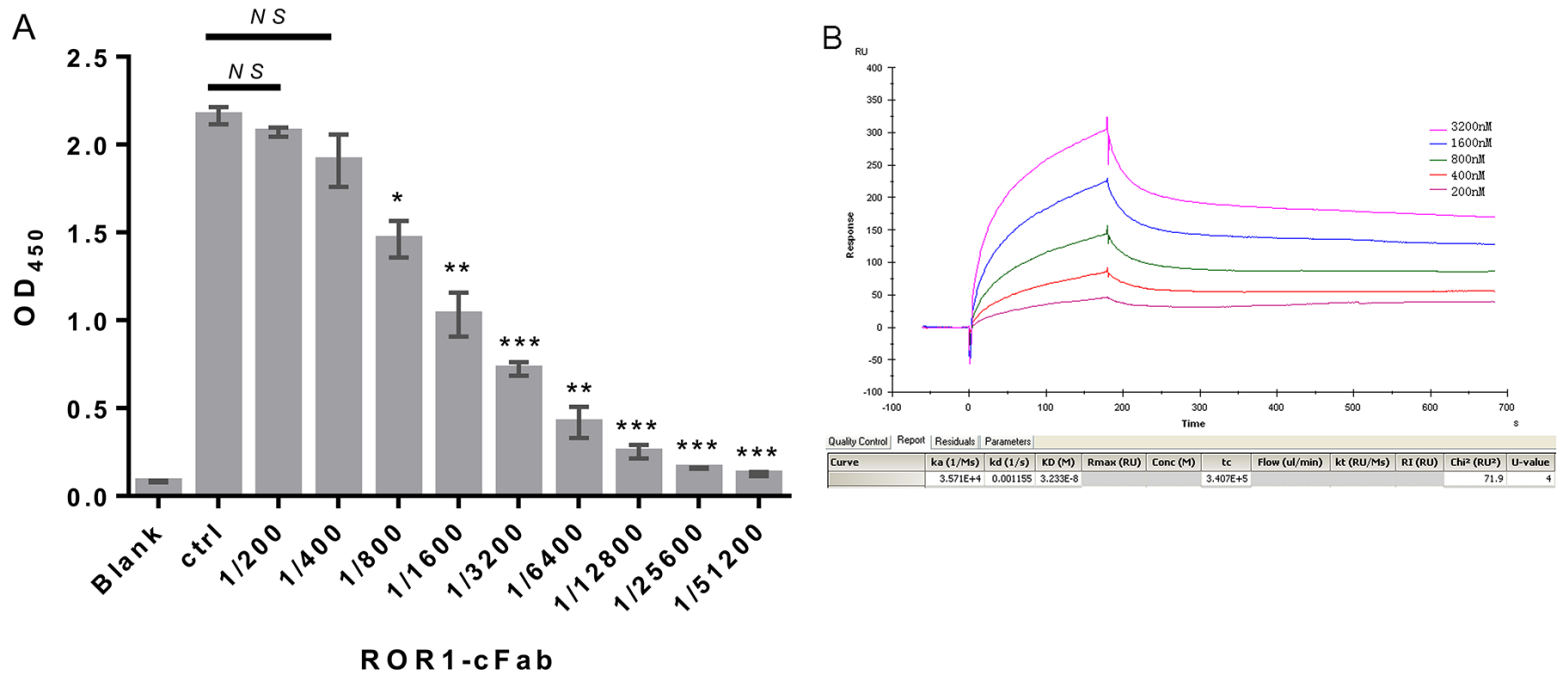
Confirmation of ROR1-cFab specificity and selectivity **(A)** ELISA. A 96-well plate was pre-coated with recombinant human ROR1 protein at a concentration of 50 ng/well. Serial dilutions of the ROR1-cFab were used as the first antibody for ELISA and the HRP-conjugated goat anti-human antibody (Fab specific) was used as the secondary antibody. Commercial anti-ROR1 antibody was used as a positive control (Ctrl). The absorbance was read at 450 nm after color development. **(B)** Surface Plasmon resonance (SPR) analysis. The ROR1 protein was diluted to 30 μg/mL in dilution buffer and then reacted in a running buffer containing serial concentrations of ROR1-cFab. Results were analyzed by the Biacore X100 software. The experiments were in triplicate and repeated at least twice. Data are shown as mean ± SD (n = 2, NS, not significant, ^*^p < 0.05, ^**^p < 0.01, and ^***^p < 0.001 compared with the control).

We assessed whether ROR1-cFab can specifically and selectively bind to human ROR1 protein using the ELISA. We first made gradient dilutions (between 20 and 0.078 μg/mL) of ROR1-cFab for ELISA and utilized a commercial anti-ROR1 antibody as the positive control and found that the ROR1-cFab specifically bound to ROR1 protein in a dose-dependent manner (Figure 2A). We further determined the binding capacity of ROR1-cFab to ROR1 protein by calculating the affinity constant that reflected the antigen-antibody reaction using the following formula: Affinity constant (KD) = dissociation constant (Kd) ÷ binding constant (Ka) according to a previous study [34]. Data from Biacore X100 SPR analysis showed that an affinity constant of ROR1-cFab of 3.233 × 10^-8^ (Figure 2B). This finding indicated that ROR1-cFab could selectively and effectively bind to the ROR1 antigen.

**Figure 3:**
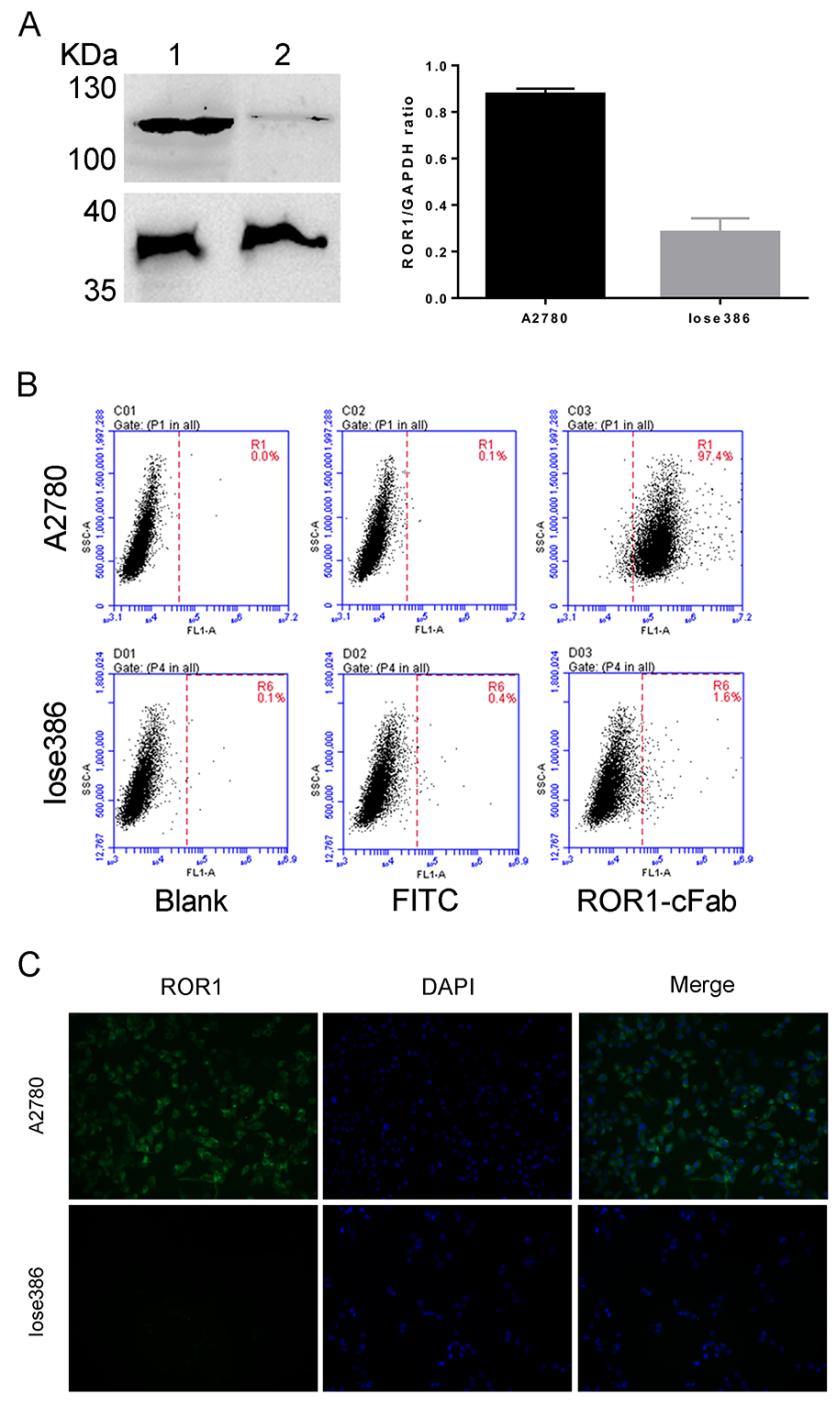
Assessment of the ROR1-cFab specificity in ovarian cancer cells **(A)** Western blot. It detected the level of ROR1 expression in ovarian cancer cells. Lane 1: supernatant of A2780 cell lysate; Lane 2: supernatant of Iose386 cell lysate. **(B)** Flow cytometry. Ovarian cancer A2780 and Iose386 cells were treated with or without ROR1-cFab for 1h and then incubated with a goat anti-human IgG (Fab specific)-FITC antibody for 1 h in the dark. The cells were then subjected to flow cytometry analysis of fluorescence intensity. **(C)** Immunofluorescence staining of ROR1. Ovarian cancer cells (A2780 and Iose386) were first grown and immunostained with ROR1-cFab. The experiments were in triplicate and repeated at least once.

In addition, we performed Western blot to determine the level of ROR1 protein in ovarian cancer A2780 and Iose386 cell lines. As shown in Figure 3A, A2780, a ROR1-positive cell line, displayed ROR1 expression, whereas Iose386, a ROR1-negative cell line, showed no expression when the ROR1-cFab was used as an antibody for Western blotting. Moreover, we also performed flow cytometry to further analyze the binding ability of ROR1-cFab to ROR1-positive A2780 cells and Iose386 cells used as a negative control. We found that ROR1-cFab-treated A2780 cells were isolated from untreated cells, but there was no apparent difference in ROR1-cFab-treated and untreated Iose386 cells (Figure 3B), suggesting that ROR1-cFab was able to selectively bind to ROR1-positive cells, but not to ROR1-negative cells.

Again, we performed immunofluorescence to visualize whether ROR1-cFab can intuitively bind to the cell surface. Our data revealed that ROR1-cFab-immunostained A2780 cells were visualized under a fluorescence microscope but staining was not visualized on ROR1-negative Iose386 cells (Figure 3C). Taken together, these results suggested that ROR1-cFab possesses a high affinity and specificity for ROR1.

### Inhibition of ovarian cancer cell malignant behaviors by the chimeric monoclonal antibody ROR1-cFab *in vitro*

**Figure 4:**
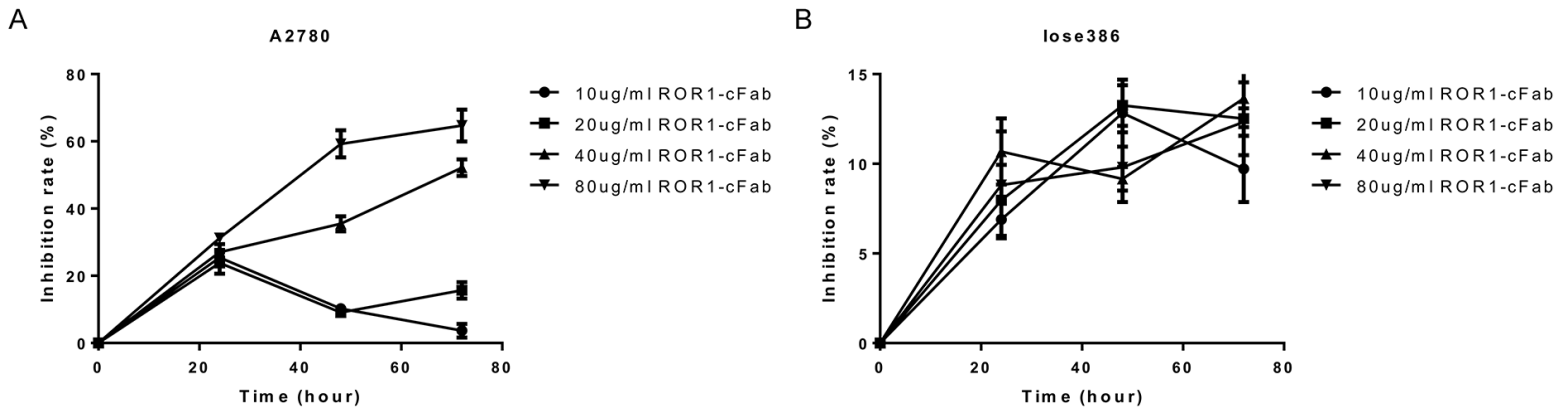
Cell viability CCK8 assay (A) A2780 cells and (B) Iose386 cells were grown and treated with different doses of ROR1-cFab for up to 72 h and then subjected to CCK8 assay.

After obtaining and verifying the properties of ROR1-cFab, we assessed its antitumor activity in ovarian cancer cell lines. We first assessed the effect of ROR1-cFab on the regulation of tumor cell viability using the CCK8 assay. In A2780 cells, when the concentration of ROR1-cFab reached to 10 μg/mL, the inhibition efficiency increased at 24 h, but decreased at 48 h and 72 h; when the concentration of ROR1-cFab was 20 μg/mL, the inhibition efficiency was increased at 24 h, decreased at 48h but increased again at 72 h; data also showed that when the concentration of ROR1-cFab was 40 and 80 μg/mL, respectively, the inhibition efficiency was increased in a time-dependent manner (approximately 50% at 40 μg/mL at 72 h and approximately 67% at 80 μg/mL at 72h; Figure 4A, 4B). Thus, we chose an incubation concentration of ROR1-cFab of 40 μg/mL in the following experiments.

**Figure 5:**
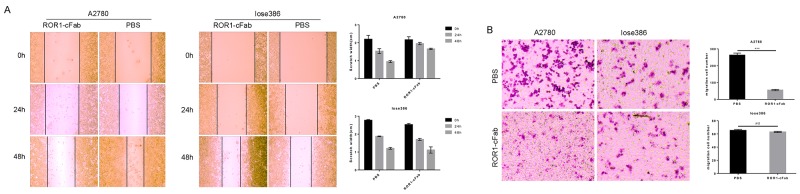
Antitumor activity of ROR1-cFab in ovarian cancer cells **(A)** Wound healing assay. Tumor cells were grown and treated with 40 μg/mL of ROR1-cFab for up to 48 h and then subjected to wound healing assay. **(B)** Transwell migration assay. Tumor cells were grown and treated with 40 μg/mL of ROR1-cFab for up to 24h and then subjected to Transwell migration assay. Data are shown as mean ± SD (n = 3, NS, not significant, ^*^p < 0.05, ^**^p < 0.01, and ^***^p < 0.001 compared with the control).

Furthermore, a tumor cell wound healing assay showed that ROR1-cFab blocked tumor cell migration; migration capacities were 0.00%, 6.98%, and 23.26% after incubation with 40 μg/mL ROR1-cFab for 0, 24, and 48 h, respectively, compared with PBS-treated A2780 cells (0.00%, 22.33% and 53.49%, respectively; Figure 5A). However, suppression of cell migration by ROR1-cFab was not apparent when Iose386 cells were used in the assay (0.00%, 32.81% and 56.52%, respectively), as compared with PBS-treated cells (0.00%, 32.13% and 56.68%, respectively; Figure 5A). In addition, we conducted a tumor cell Transwell migration assay to further investigate the effect of ROR1-cFab on ovarian cancer cells *in vitro*. Our data showed that ROR1-cFab significantly reduced migration of A2780 cells by approximately 81.4% after incubation with 40 μg/mL ROR1-cFab for 24 h. Treatment of Iose386 cells with the same concentration of ROR1-cFab did not reduce migration (Figure 5B). Thus, our results implied that the ROR1-cFab could effectively inhibit tumor cell migration in a time-dependent manner.

**Figure 6:**
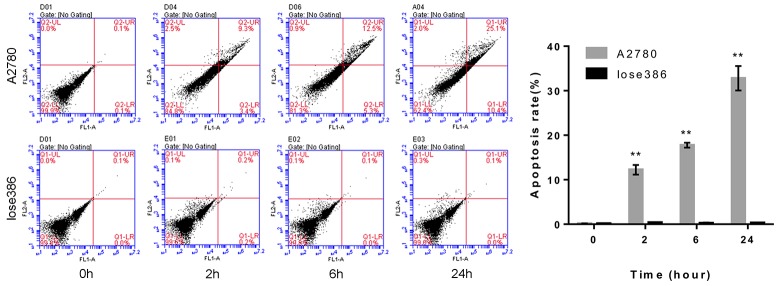
Flow cytometric apoptosis assay Tumor cells were grown and treated with 40 μg/mL of ROR1-cFab for up to 24h and then subjected to flow cytometric apoptosis assay. As a ROR1-negative cell line, Iose386 showed no difference in these assays. Data are shown as mean ± SD (n = 3, NS, not significant, ^*^p < 0.05, ^**^p < 0.01, and ^***^p < 0.001 compared with the 0h group).

Next, we assessed the pro-apoptotic effect of the ROR1-cFab on ovarian cancer cells using flow cytometric Annexin V-FITC/PI apoptosis assay. We found that continuous exposure to ROR1-cFab resulted in an increased rate of apoptosis of A2780 cells with 12.7% for 2 h, 17.8% for 6 h and 35.5% for 24 h. These effects were not apparent in Iose386 cells (Figure 6). Taken together, our current study revealed that ROR1-cFab could effectively reduce malignant behavior of ovarian cancer A2780, but not Iose386 cells, indicating that ROR1 is the *in vitro* target of ROR1-cFab antibody.

## DISCUSSION

In the current study, we produced a novel chimeric monoclonal Fab antibody against human ROR1 protein using an *in vitro* protein expression and cell fusion technique for the generation of a monoclonal antibody. We then assessed antitumor effects of the generation antibody, ROR1-cFab, on ovarian cancer cell lines. We found that: 1) ROR1-cFab possesses a high affinity and specificity for ROR1 and 2) ROR1-cFab was able to effectively reduce malignant behaviors of ROR1-positive ovarian cancer A2780 cells, but not in ROR1-negative Iose386 cells *in vitro*. Our future studies will further verify the antitumor activity in ovarian cancer *in vivo*.

Different DNA recombination technologies are useful to develop and generate human antibodies, which are widely applied for both the diagnosis and treatment of various diseases. Among them, cell fusion technology has the advantages of both high efficiency and selectivity. Previous studies have reported the successful development and production of chimeric monoclonal antibodies using this methodology [35–38]. Chimeric monoclonal antibodies contain IgG and Fab fragments and these fragments possess a high validity to translocate into targeted tissues or cells with a high concentration and efficiency.

In the current study, we combined cell fusion and *in vitro* construction of ROR1-cFab fragments to separate a chimeric anti-ROR1 monoclonal Fab (ROR1-cFab) to identify and recognize recombinant ROR1 protein. This approach was performed by repeated screening with pre-coated recombinant human ROR1 protein in 96-well plates to guarantee the efficiency of specific ROR1 binding. After three rounds of sub-clone affinity screening, we selected five positive fusion cell clones from a total of 31 candidate clones with a strong ELISA positive signal and specificity to bind to ROR1 protein antigen. In parallel, we also successfully constructed the prokaryotic expression vector pETDuet-ROR1-cFab and expressed and verified the two different heavy and light chains of fragments in order to ensure the production of a stable Fab antibody. A previous study showed that such an approach is reliable and efficient [39]. We then evaluated and verified adequate expression and purification of ROR1-cFab using SDS-PAGE gels for Coomassie blue staining and Western blotting. To examine the specificity and selectivity of this antibody, we employed other techniques such as ELISA, affinity analysis, FACS, and immunofluorescence staining to determine the binding capacity of ROR1-cFab to recombinant human ROR1 antigen and ROR1 protein on the surface of ovarian cancer cells. Therefore, our current results illustrated that such an antibody engineering process did not affect or alter the specificity of this chimeric anti-ROR1 Fab.

Furthermore, we assessed the antitumor activity of this ROR1-cFab in ovarian cancer cells, our *in vitro* model of preclinical cancer therapy. In this model, we selected two ovarian cancer cell lines – both ROR1-positive and negative cell lines – allowing us to further explore the specificity and selectivity of ROR1-cFab. Our cell viability CCK8 data demonstrated that if the ROR1-cFab concentration was equal or greater than 40μg/mL, ROR1-cFab treatment was able to efficiently reduce the viability of ROR1-positive ovarian cancer cells in a time-dependent manner. Our wound healing and Transwell migration assays revealed significant inhibition of migration ability of ROR1-positive ovarian cancer cells at a dose of 40μg/mL ROR1-cFab for 24 or 48h. Furthermore, an Annexin V-FITC/PI apoptosis assay demonstrated that ROR1-cFab also induced apoptosis of ROR1-positive A2780 cells in a time-dependent manner. However, ROR1-negative ovarian cancer lose386 cells failed to undergo apoptosis upon treatment with ROR1-cFab. Thus, these data clearly indicate that ROR1-cFab can selectively and specifically block functions of ROR1 in ovarian cancer cells and effectively reduce tumor cell malignant behaviors. In future studies, we aim to examine the *in vivo* effects of ROR1-cFab.

Previous studies have demonstrated that ROR1 antibody or knockdown of ROR1 expression can effectively reduce proliferation of various types of cancer cells, such as chronic lymphocytic leukemia (CLL) cells, ovarian cancer cells, and lung adenocarcinoma cells [22–24]. Molecularly, a previous study also highlighted that miR-27b-3p could activate cell proliferation, regulate colony formation and promote tumorigenicity by targeting ROR1 in gastric cancer cells [40]. Further data showed the c-Src/STAT3 signaling pathway may be involved in miR-27b-3p-ROR1-mediated tumor cell proliferation [40]. Another previous study demonstrated that STAT3 may bind to the ROR1 promoter region after constitutive STAT3 phosphorylation and activation [41], while other studies have shown that Wnt5a signaling might be one of the ligands proving to induce ROR1 expression [5, 42, 43]. In addition, it has also been reported that ROR1 may form a complex with TCL1, in order to activate AKT and in turn to induce expression of cell growth-related genes and reduce expression of apoptosis-related genes [44]. However, the underlying mechanisms responsible for ROR1 action and promotion of ovarian cancer malignant phenotypes remain to be defined, which is also a limitation to our current study. Our current study is a proof-of-principle approach to assess the antitumor activity of the generated ROR1 antibody. So there are some limitations in the current study; for example, we have had a ROR1-negative cell line as a control, but we should have also used normal ovarian epithelial cells as a control since ROR1 could be expression at a low level in some normal cells; thus, such a cell line could be used as control of this antibody-induced cell toxicities. Moreover, in future studies, we will investigate the potential mechanisms and biological behavior of ROR1 in ovarian cancer *in vivo*.

In conclusion, in our current study, we successfully developed and produced a chimeric anti-ROR1monoclonal Fab antibody (ROR1-cFab), which could selectively and specifically bind to ROR1 protein with a high affinity. We also demonstrated that ROR1-cFab was able to specifically inhibit ovarian cancer cell growth and induce apoptosis in ROR1 expressing cells, suggesting that this antibody could be further investigated as an appropriate and promising therapeutic strategy for ROR1-positive human cancers.

## MATERIALS AND METHODS

### Animals and immunization with ROR1 protein

Our animal study protocol was approved by the Ethics Committee of Nanjing Medical University, China, and we strictly followed the Guiding Principles of the Animal Care and Use of Nanjing Medical University, China. Eight-week-old female BALB/c mice were purchased from SLRC Laboratory Animals (Shanghai, China) and maintained in three animals per cage and received food and water *ad libitum* in Nanjing Medical University Experimental Animal Center (Nanjing, China) with thick sawdust bedding at standard room temperature (22 ± 2°C), under conditions of a 12/12-hr reversed light-dark cycle (7:00 am-7:00 pm). Mice were acclimatized to the laboratory environment for 3–5 days before our experiments.

Three female mice were subcutaneously immunized with soluble recombinant human ROR1 protein (50 μg per mouse) emulsified with complete Freund’s adjuvant (Sigma Chemicals, St. Louis, MO, USA). Recombinant human ROR1 protein (ECD, His Tag) was purchased from Sino Biological Inc. (Beijing, China). Two weeks later, mice were treated with an intraperitoneal injection of ROR1 protein emulsified with incomplete Freund’s adjuvant (Sigma Chemicals, St. Louis, MO, USA) for the second immunization. After two weeks, mice were subcutaneously injected with the same formulation of ROR1 protein for the third immunization. Two weeks later, sera from these mice were collected to determine the level of ROR1 protein immunity.

### Cell fusion and screening of positive fused cell clones

Splenocytes from the mice with the highest immune titers of ROR1 antibodies were collected and fused with myeloma cells (detailed below) using Polyethylene Glycol 1500 (PEG 1500; Roche, Indianapolis, IN, USA). Fused cells were then seeded in 96-well plates and cultured in Dulbecco’s Modified Eagle’s Medium (DMEM, GIBCO BRL, Gaithersburg, MD, USA) supplemented with 10% fetal bovine serum (FBS; GIBCO BRL), 1% penicillin (100 U/mL) and 1% streptomycin (GIBCO BRL) for approximately 11 days. Following this, 50 μL supernatant from each well with replicating fused cells was collected and added into a fresh 96-well plate that was pre-coated with 50 ng recombinant human ROR1 protein (Sino Biological Inc., Beijing, China). After incubation for 1h at room temperature and washing three times in PBS-Tween 20 (PBST), 50 μL of horseradish-peroxidase (HRP)-conjugated rabbit anti-mouse IgG antibody (Sigma Chemicals, St. Louis, MO, USA) solution was added into each well at a dilution of 1:5,000 in blocking buffer and plates were incubated for 1h and subsequently measured with a spectrophotometer (Thermo Electron Corporation, MA, USA) at 450 nm. Fusion cells with high immune titers were picked up and subjected to three rounds of sub-clone affinity screening. Positive fusion cell clones were then selected and assessed.

### Construction of an expression vector for chimeric monoclonal antibody ROR1-cFab

Total RNA was isolated from positive fusion cell clones after three rounds of sub-clone affinity screening using Trizol reagent (Invitrogen, Carlsbad, CA, USA) and reversed transcribed into cDNA using Prime Script RT reagent (TaKaRa, Shiga, Japan) according to the manufacturers’ instructions [31]. Resulting cDNA samples were saved as templates in order to PCR amplify the variable regions of the heavy chain (V_H_) and the light chain (V_L_) with degenerate primers (Table 1). PCR products were purified with the Wizard SV Gel and PCR Clean-Up System (Promega, Madison, WI, USA) and then cloned into pMD-18T vectors, respectively. After amplification and DNA sequence confirmation, pMD-18T vectors carrying different V_H_ and V_L_ were analyzed using the VBASE2 database (http://www.vbase2.org/). Additionally, the human conserved regions of the heavy chain (C_H_1) and the light chain (C_L_) were acquired from the Barbas laboratory (Scripps Research Institute, San Diego, CA, USA) and after DNA sequence confirmation, they were combined with our newly cloned V_H_ and V_L_, respectively. The newly formed V_H_-C_H_1 and V_L_-C_L_ were used as templates for PCR amplification with HF1, HR2, and LF1, LR2, respectively, to obtain the heavy chain Fd and the light chain L. After amplification and DNA sequence confirmation, purified Fd and L were finally cloned into pETDuet-1 at NdeI/kpnI and NcoI/HindIII sites, respectively.

**Table 1:** Primers used for the construction of the ROR1-cFab gene

Primer name	DNA sequence
Heavy chain variable region forward primer	
V_H_F1	GCTGCCCAACCAGCCATGGCCCAGGTGCAGCTGGTGCAGTCTGG
V_H_F2	GCTGCCCAACCAGCCATGGCCCAGATCACCTTGAAGGAGTCTGG
V_H_F3	GCTGCCCAACCAGCCATGGCCGAGGTGCAGCTGGTGSAGTCTGG
V_H_F4	GCTGCCCAACCAGCCATGGCCGAGGTGCAGCTGKTGGAGTCTG
V_H_F5	GCTGCCCAACCAGCCATGGCCCAGGTGCAGCTGCAGGAGTCGGG
V_H_F6	GCTGCCCAACCAGCCATGGCCCAGGTGCAGCTACAGCAGTGGGG
Heavy chain variable region reverse primer	
V_H_R1	CGATGGGCCCTTGGTGGAGGCTGAGGAGACGGTGACCAGGGTTCC
V_H_R2	CGATGGGCCCTTGGTGGAGGCWGRGGAGACGGTGACCAGGGTBCC
Light chain variable region forward primer	
V_L_F1	GGGCCCAGGCGGCCGAGCTCCAGATGACCCAGTCTCC
V_L_F2	GGGCCCAGGCGGCCGAGCTCGTGATGACYCAGTCTCC
V_L_F3	GGGCCCAGGCGGCCGAGCTCGTGWTGACRCAGTCTCC
V_L_F4	GGGCCCAGGCGGCCGAGCTCACACTCACGCAGTCTCC
Light chain variable region reverse primer	
V_L_R1	GAAGACAGATGGTGCAGCCACAGT
Heavy chain variable region primer	
HF1	CATATGCAGGTGCAGCTGGTGCAGTCTG
HR1	TGGGCCCTTGGTGGAGGCGGAGACGGTGACCAGGGTTC
Light chain variable region primer	
LF1	CCATGGGCGAGCTCGTGATGACCCAG
LR1	CAGCCTTGGGCTGACCTTTTATTTCCAACTTTGTC
Constant region C_H_1 primer	
HF2	GAACCCTGGTCACCGTCTCCGCCTCCACCAAGGGCCCA
HR2	GGTACCTTAAGAAGCGTAGTCCGGAAC
Constant region C_L_ primer	
LF2	GACAAAGTTGGAAATAAAAGGTCAGCCCAAGGCTG
LR2	AAGCTTTTATGAACATTCTGTAGGGGCCACT
Heavy chain primer	
HF1	CATATGCAGGTGCAGCTGGTGCAGTCTG
HR2	GGTACCTTAAGAAGCGTAGTCCGGAAC
Light chain primer	
LF1	CCATGGGCGAGCTCGTGATGACCCAG
LR2	AAGCTTTTATGAACATTCTGTAGGGGCCACT

### Expression and purification of chimeric monoclonal antibody ROR1-cFab

The pETDuet-1 carrying the heavy chain Fd and the light chain L were transformed into *E. coli* BL21 (TRANS, Beijing, China) and *E. coli* were induced at 16 or 37°C with 1mmol/L isopropyl β-D-thiogalactopyranoside (IPTG) overnight. We found that recombinant E. *coli* had higher expression levels of Fab fragments at 16°C than that at 37°C (data not shown). Thus, a single clone was then inoculated in LB medium supplemented with 100 μg/mL ampicillin and stimulated by 1 mmol/L isopropyl β-D-thiogalactopyranoside (IPTG) at 16°C for 12h. After ultrasonic cytolysis and centrifugation, soluble ROR1-cFab in the supernatant was separated and purified using Protein L affinity column from GE Healthcare (Piscataway, NJ, USA) according to the manufacturer’s instructions. The bacteria lysate, sonicated supernatant, and the ROR1-cFab protein were dissociated in 10% sodium dodecyl sulfate-polyacrylamide gel electrophoresis (SDS-PAGE) gel and stained with Coomassie blue and analyzed using Western blotting, respectively.

### Western blot

Expression of ROR1-cFab in *E. coli* BL21 was analyzed by using Western blotting as described previously [32]. In brief, proteins from *E. coli* were purified with Protein L affinity column from GE Healthcare, the concentration of which was measured using the bicinchoninic acid protein assay kit (Thermo, Waltham, MA, USA). Protein samples were then dissociated in 10% SDS-PAGE gels and transferred onto nitrocellulose membranes (Bio-Rad, Hercules, CA, USA). For Western blotting, membranes were incubated in 5% non-fat milk in Tris-based saline-Tween 20 (TBS-T) for 2h at the room temperature and then with horseradish peroxidase (HRP)-conjugated goat anti-human Fab specific antibody (Santa Cruz Biotechnology, Santa Cruz, CA, USA) at a dilution of 1:1000 for 1 h at the room temperature. Similarly, ROR1 expression was analyzed using Western blot with our newly developed anti-ROR1 antibody in ovarian cancer cell lines (A2780 and Iose386).

### Enzyme-linked immunosorbent assay (ELISA)

Non-competitive ELISA was employed to identify the affinity of ROR1-cFab. Briefly, serial dilutions of ROR1-cFab were added into 96-well plates that were pre-coated with 50 ng recombinant human ROR1 protein(Sino Biological Inc., Beijing, China) per well. After incubation and washing with PBST, HRP-conjugated goat anti-human Fab specific antibody(Santa Cruz Biotechnology, Santa Cruz, CA, USA) was added into each well of the plates, while a commercial anti-human ROR1 antibody (Abcam, Cambridge, MA, USA) was used as a positive control. The absorbance value at 450 nm was then detected using a spectrophotometer (Thermo Electron Corporation).

### Surface plasmonresonance (SPR) analysis of chimeric monoclonal antibody ROR1-cFab

The binding affinity of the antibody was also analyzed by the Biacore X100 SPR system (GE Health, Sweden). We followed the isoelectric point of the ROR1 protein and the optimization of coupling conditions provided by the manufacturer to select sodium acetate as the dilution buffer for the assay. After dilution to 30 μg/mL, the ROR1 protein was coupled to the CM5 chip and the coupling level was preset at 1,500 RU, while the injection time was preset to 180 s and the dissociation time was preset to 15 min. We used 50 mM Gly-HCl (pH 1.7) as the regeneration buffer. The ROR1 protein reacted with the serial concentrations of ROR1-cFab, which was determined using the Biacore X100 SPR system as the binding affinity of the antibodies.

### Cell lines and culture

A human serous cystic adenocarcinoma ovarian cancer cell line A2780 and human ovarian epithelial cell line Iose386 were obtained from The Cell Bank of Chinese Academy of Sciences (Shanghai, China). Mouse myeloma cells were conserved by our laboratory. Lose386 cells were cultured in RPMI-1640(GIBCO BRL, Gaithersburg, MD, USA), while A2780 and mouse myeloma cells were cultured in DMEM (GIBCO BRL, Gaithersburg, MD, USA) and both were supplemented with 10% FBS (GIBCO BRL), 1% penicillin (100 U/mL) and 1% streptomycin (100 μg/mL) in a humidified atmosphere with 5% CO_2_ at 37°C.

### Fluorescence-activated cell sorting (FACS)

Ovarian cancer A2780 and Iose386 cell lines were incubated with the ROR1-cFab for 1h and then incubated with a goat anti-human IgG (Fab specific)-FITC antibody (Sigma Chemicals, St. Louis, MO, USA) for 1 h. After being washed in PBS, the fluorescence intensity of cells was detected by the LSRII flow cytometer (BD Biosciences, San Jose, CA, USA).

### Immunofluorescence assay

Ovarian cancer A2780 and Iose386 cell lines were seeded into 6- well plates and grown to reach approximately 80% confluency. Next, cells were fixed in 4% paraformaldehyde and subsequently blocked in 5% non-fat milk for 1 h and then with the ROR1-cFab for 1 h at the room temperature and further with a goat anti-human IgG (Fab specific)-FITC antibody (Sigma Chemicals, St. Louis, MO, USA) for 1 h. 4′-6-diamidino-2-phenylindole (DAPI, Biotium, Hayward, CA, USA) was used to stain cell nuclei. Cells were analyzed under a fluorescence microscope (Zeiss, Jena, Germany).

### Cell viability CCK8 assay

The changed cell viability after treatment with our newly developed anti-ROR1 antibody was assayed using the CCK8 Kit (Dojindo, Rockville, MD, USA). In brief, ovarian cancer A2780 and Iose386 cell lines were seeded into a 96-well plate in triplicate and then treated with serial concentrations of our newly-developed anti-ROR1 antibody for 24 h, 48 h, or 72 h. After, cell cultures were treated with the CCK8 reagent and further cultured for 2 h. The optical density at 450 nm was measured with a spectrophotometer (Thermo Electron Corporation, MA, USA). The percentage of the control samples of each cell line was calculated thereafter.

### Tumor cell wound healing assay

Ovarian cancer A2780 and Iose386 cell lines were seeded into 24-well plates and grown to reach to 90% confluency. A scratch was then made across a monolayer of cells using a 200 μL pipette tip and treated with 40 μg/mL ROR1-cFab for 0, 24, and 48 h. Image Pro Plus software was employed to calculate wound closing rate according to the following formula: n h (n hour) migration rate= (the distance from the edge of the 0 h- the distance from the edge of the n h)/ the distance from the edge of the 0 h.

### Tumor cell transwell migration assay

To evaluate cell migration capacity, ovarian cancer A2780 and Iose386 cell lines were seeded into Transwell inserts (Corning Life Sciences, Tewksbury, MA, USA) at a density of 4 × 10^4^ cells/well and treated with 40μg/mL ROR1-cFab. The bottom of the Transwell was filled with 10% FBS and Transwell plates were cultured for 24 h. At the end of each experiment, cells remaining at the top surface of the Transwell inserts were removed using cotton swabs, whereas cells which migrated onto the inverse side of the Transwell inserts were fixed with 4% paraformaldehyde and subsequently stained with 0.1% crystal violet stain (Sigma Chemicals, St. Louis, MO, USA) and counted for five randomly-selected fields under an inverted microscope (Olympus, Tokyo, Japan). The mean migration rate was calculated for % of control in each cell line.

### Flow cytometric cell apoptosis assay

Ovarian cancer A2780 and Iose386 cell lines were grown and treated with 40 μg/mL ROR1-cFab for 0, 2, 6, and 12 h, and then collected and washed in PBS to prepare a single cell suspension in a binding buffer from the Annexin V-FITC/PI apoptosis kit (BD Biosciences, San Jose, CA, USA). According to the manufacturer’s protocol, we labeled cells with Annexin V-FITC/PI and measured them using the LSRII flow cytometer (BD Biosciences, San Jose, CA, USA). The percentage of cell apoptosis vs. control was then calculated for each experiment.

### Statistical analysis

The data were summarized as mean ± SD and the statistical significance of group comparisons was analyzed using Student’s *t* test. A *p* value<0.05 was deemed as statistically significant.
